# Adequate tissue sampling for the assessment of pathological tumor regression in pancreatic cancer

**DOI:** 10.1038/s41598-021-86152-y

**Published:** 2021-03-22

**Authors:** Masanao Yokohira, Minoru Oshima, Keiko Yamakawa, Juanjuan Ye, Yuko Nakano-Narusawa, Reiji Haba, Yuki Fukumura, Kenichi Hirabayashi, Hiroshi Yamaguchi, Motohiro Kojima, Keiichi Okano, Yasuyuki Suzuki, Yoko Matsuda

**Affiliations:** 1 Oncology Pathology, Department of Pathology and Host-Defense, Faculty of Medicine, Kagawa University, 1750-1, Ikenobe, Miki-cho, Kita-gun, Kagawa, 761-0793 Japan; 2 Department of Gastroenterological Surgery, Faculty of Medicine, Kagawa University, Kagawa, Japan; 3 Department of Diagnostic Pathology, Faculty of Medicine, Kagawa University, Kagawa, Japan; 4 Department of Human Pathology, School of Medicine, Juntendo University, Tokyo, Japan; 5 Department of Pathology, Tokai University School of Medicine, Kanagawa, Japan; 6 Department of Pathology, Saitama Medical University, Saitama, Japan; 7 Division of Pathology, Exploratory Oncology Research and Clinical Trial Center, National Cancer Center, Kashiwa, Japan

**Keywords:** Pancreatic cancer, Outcomes research

## Abstract

Standardized pathological evaluation of the regression assessment of neoadjuvant pancreatic cancer is necessary to improve prognostication and compare treatment outcomes in clinical trials. However, appropriate tissue sampling from surgically resected pancreatic cancer after neoadjuvant therapy has not been elucidated. We compared the tumor regression scores in the largest cancer slide determined macroscopically or histologically. We reviewed all slides and macroscopic photos of cut surfaces from resected pancreas of patients treated with neoadjuvant chemotherapy (n = 137; chemoradiotherapy or chemotherapy). The tumor regression scores (the Evans, College of American Pathologists, Japanese Pancreas Society grading systems, and Area of Residual Tumor [ART] score) were evaluated for the largest tumor slide determined by macroscopy or histologically as well as all slides from the resected pancreas. The largest cancer slides determined macroscopically and histologically were discrepant in 26% of the cases. Cancer cells were not detected in the largest macroscopically defined cut slides in 3%. Only ART scores assessed in the largest histological slides displayed significant difference in overall survival. We recommend obtaining the largest histological slides to provide adequate assessment for regression of neoadjuvant-treated pancreatic cancer. Sufficient sampling to detect the largest histological slides would be mandatory.

## Introduction

Despite advances in diagnostics and therapeutics, the prognosis of pancreatic cancer remains poor, with an overall 5-year survival rate of approximately 7%^1^. Surgery remains the only curative therapy for patients with pancreatic cancer. However, the effect of pancreatectomies on patients' quality of life and long-term survival remains contentious. Additionally, the clinical benefit of traditional upfront surgery has been shown to be limited, and more than 90% of patients relapse and die due to their disease after surgery^2^. Recently, neoadjuvant therapies for resectable or borderline-resectable pancreatic cancers, which improve the control of local tumors and micrometastases, have been reported to improve clinical outcomes and are currently introduced as standard therapy^3–5^.

Pathology assessments on tumor regression are considered to be important to predict patient outcomes of pancreatic cancer when receiving neoadjuvant therapy^6^. Several grading systems, such as Evans^7^, the College of American Pathologists (CAP)^8^, and the Japanese Pancreas Society (JPS)^9^, are currently used to assess tumor regression. Recently, we proposed the area of residual tumor (ART)-based pathological assessment, which provides more robust prognostication and objective assessment^10–12^. However, thus far, there have been no standardized techniques for tissue sampling in pancreatic cancer after neoadjuvant therapy. The CAP protocol suggests that the gross measurement of tumor size has to be validated by microscopic examination and in order to validate histologically the tumor size, pathologist should sample the largest tumor area/tumor bed as well as making a generous sampling of the adjacent parenchyma/adipose tissue. When no tumor is visible, the entire specimen should be sampled to rule out any microscopic residual carcinoma. Even in the CAP classification with such detailed criteria, the actual evaluation method varies depending on the pathologist. Therefore, current sampling of specimens can vary among pathologists and institutions and may cause inter-institutional inconsistency.

Generally, residual tumors are less defined than untreated tumors, and macroscopic identification of viable tumor area is often difficult. Therefore, it is unclear whether tissue sampling from the largest macroscopically defined slides is sufficient for adequate histological assessment. More standardized pathological preanalytic procedures for neoadjuvant pancreatic cancer specimens are necessary to improve prognostication, which will allow us to obtain real-world regression data without bias. In the present study, we analyzed the prognostic predictability of several tumor regression score assessed in the largest macroscopically defined slides, the largest histological slides, and all slides from whole-tumor slices to establish a more standardized sampling method for the pancreatic cancer tissue that received preoperative therapy.

## Results

**Figure 1:**
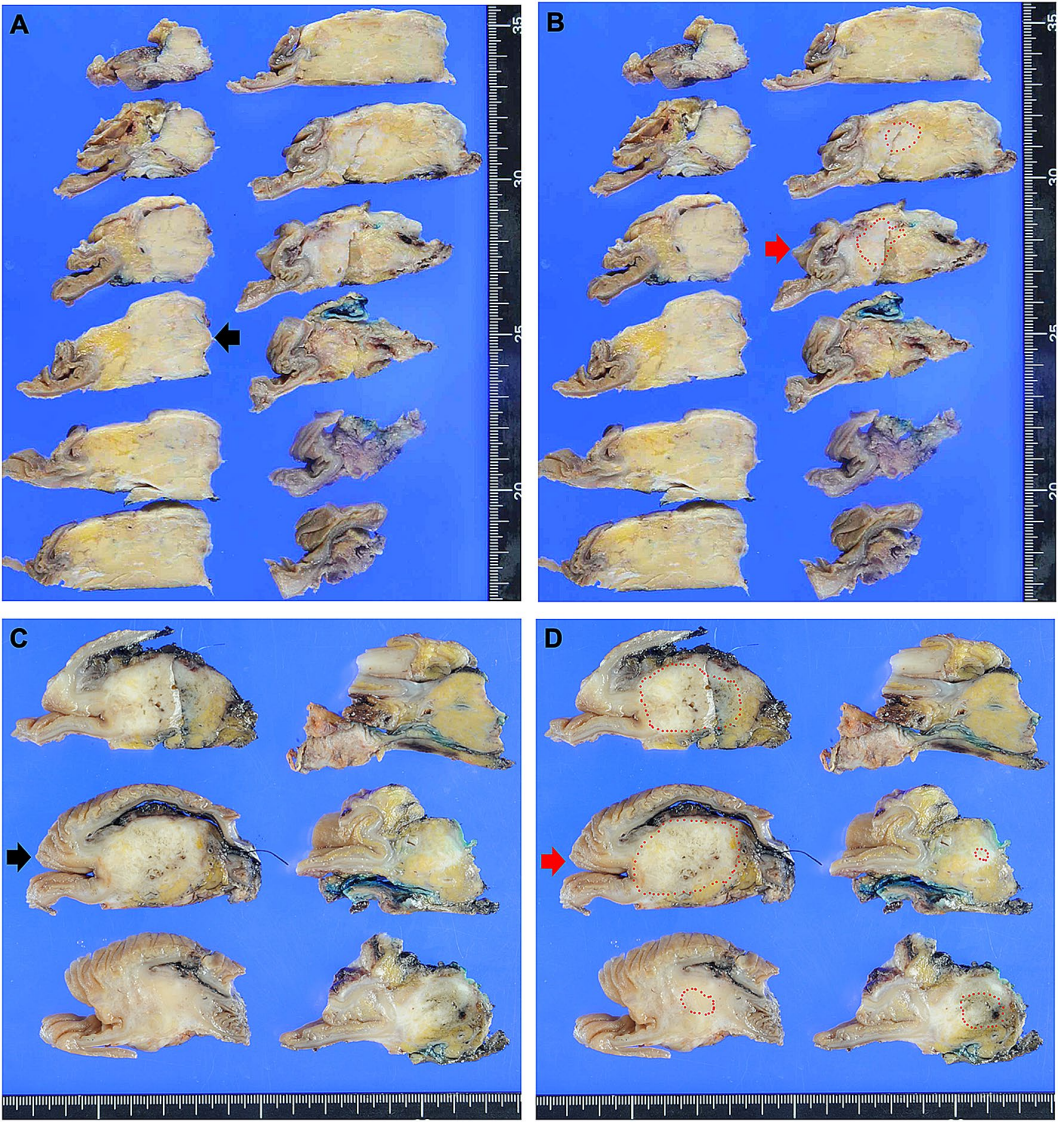
Images showing the areas that are easy or difficult to identify as the cancer area. (**A**), (**B**), (**C**), and (**D**) are the same areas of the pancreas, respectively. (**A**) and (**B**) are easy to identify, while (**C**) and (**D**) are difficult to identify. The black arrows show the largest cancer area determined macroscopically, and the red arrows show the largest cancer area determined histologically. Circles with a red dotted line indicate the histologic cancer area.

Most patients showed poor or no response (Evans I or IIa, 96%; JPS 1, 92%; CAP 3, 89%; Table 1). One patient (0.7%) had a pathological complete response (Evans IV, JPS 4, CAP 0). The largest macroscopically defined cut surface and largest histological slide were concordant in 74% of cases (n = 101), while those in 26% of cases (n = 36) were not (discrepant cases, Table 1). Macroscopically, the coincidence cases were well-circumscribed solid cancers (Fig. 1A, B). The discrepant cases were poorly marginated cancers accompanied by fibrosis in the pancreas (Fig. 1C, D). Furthermore, in 3% of patients (n = 4), viable tumor cells were not obtained in the largest macroscopically defined cut surfaces.

**Table 1:** Clinicopathological characteristics of patients with pancreatic cancer that underwent neoadjuvant therapy, showing the coincidence or discrepancy between macroscopy and histology.

	Total		Coincidence		Discrepancy	
Number of patients, n (%)	137		101 (74)		36 (26)	
**Age, y**						
Mean, range	69, 49–87		70, 49–87		66, 49–84	
≥ 70, n (%)	64 (47)		53 (39)		11 (8)	
Sex, male, n (%)	86 (63)		67 (49)		19 (14)	
**Preoperative treatment, n (%)**						
CRT	85		68 (79)		17 (21*)	
CT	52		33 (65)		19 (35)	
**Vascular invasion, n (%)**						
Positive	18		14 (78)		4 (22)	
Negative	119		87 (73)		32 (27)	
**Perineural invasion, n (%)**						
Positive	109		82 (75)		27 (25)	
Negative	28		19 (68)		9 (32)	
Resection margin negative, n (%)	119 (87)		90 (66)		29 (21)	
**Stage (UICC 8th), n (%)**						
0	2		2 (100)		0 (0)	
IA	7		4 (57)		3 (43)	
IB	12		9 (75)		3 (25)	
IIA	48		37 (77)		11 (23)	
IIB	51		35 (69)		16 (31)	
III	14		13 (93)		1 (7)	
IV	3		1 (33)		2 (67)	
Cancer size, mean ± S.D., mm	30.0 ± 15.9		28.9 ± 15.9		32.9 ± 15.9	
**Cancer location, n (%)**						
Head	93		66 (71)		27 (29)	
Body/Tail	44		35 (80)		9 (20)	
**Pathological differentiation, n (%)**						
Well	70		52 (74)		18 (26)	
Moderately	50		35 (70)		15 (30)	
Poorly	12		9 (75)		3 (25)	
Unclassified	5		5 (100)		0 (0)	
**Evans, n (%)**						
I, IIa	131		98 (75)		33 (25)	
IIb, III, IV	6		3 (50)		3 (50)	
**JPS, n (%)**						
1	126		95 (75)		31 (25)	
2–4	11		6 (55)		5 (45)	
**CAP, n (%)**						
0–2	15		14 (93)		1 (7*)	
3	122		87 (71)		35 (29)	
**ART, n (%)**						
0–3	55		43 (78)		12 (22)	
4	82		58 (71)		24 (29)	
**Necrosis, n (%)**						
Low, ~ 19%	70		58 (83)		12 (17*)	
High, 20% ~	67		43 (64)		24 (36)	
**T/S ratio, n (%)**						
Low, ~ 39%	95		71 (75)		24 (25)	
High, 40% ~	42		30 (71)		12 (29)	

CRT, chemoradiation; CT chemotherapy.; UICC, Union for International Cancer Control; T/S ratio, tumor stroma ratio.

*P < 0.05 versus the coincidence group.

Evans, JPS, CAP and ART scores were determined by all slides.

Discrepant cases were found more frequently in the CT group (67%) than in the CRT group (47%, P < 0.05, Table 1). The discrepant cases showed more robust tumor regression by CAP and showed more frequent necrosis than the concordant cases (P < 0.05, respectively; Table 1). Compared to the score from largest macroscopically defined cut surfaces, each score determined by the largest histological slides showed a higher correlation to those determined by all slides from whole-tumor slices (Table 2).

**Table 2:** Multivariate correlation values for tumor regression grades from slides of macroscopic defined largest tumor and of largest histological tumor versus all slides.

	All	Macroscopy	Histology
Evans	1.0000	0.8473	0.8815
JPS	1.0000	0.9486	0.9641
CAP	1.0000	0.8546	0.9721
ART	1.0000	0.8457	0.9389

**Figure 2:**
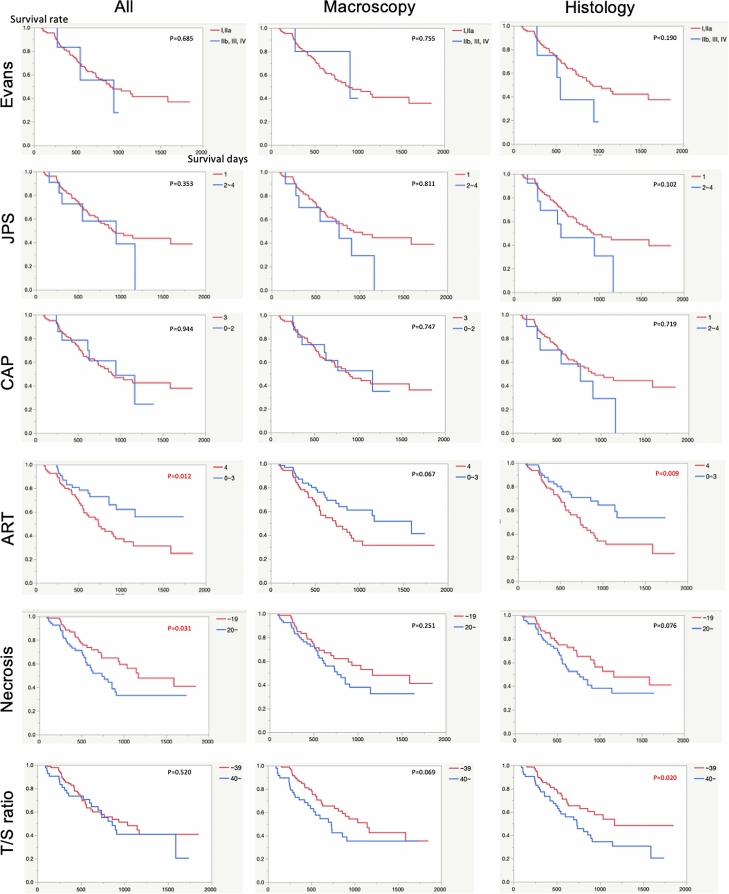
Overall survival of patients receiving neoadjuvant therapy. Kaplan–Meier analysis was performed to analyze the relationship between overall survival and clinicopathological features. All evaluation using all slides of the pancreas; Macroscopy, evaluation using the largest cancer cut surface determined macroscopically; Histology, evaluation using the largest cancer slide determined histologically.

Next, prognostic values among regression grades were assessed in histological and largest macroscopically defined slides. Only the ART scores determined by the largest histological slides were significant in terms of overall survival (P = 0.012 and P = 0.009, respectively; Fig. 2 and Table 3). The Evans, JPS, and CAP scoring systems were not associated with overall survival in both largest histological and macroscopically defined slides. Furthermore, cases with high necrosis determined by all slides from whole-tissue slices showed a better prognosis than cases with low necrosis (P = 0.0031; Fig. 2 and Table 3). Cases with a low T/S ratio, determined histologically by the largest slides, showed a better prognosis than cases with a high T/S ratio (P = 0.020; Fig. 2 and Table 3).

**Table 3:** P value of the log-rank test for overall survival after surgery.

	All			Macroscopy			Histology		
	No. (%)	Survival days (mean ± SD)	P value	No. (%)	Survival days	P value	No. (%)	Survival days (mean ± SD)	P value
**Evans**									
I, IIa	131 (96)	993.9 ± 55.8	0.685	128 (93)	983.1 ± 56.4	0.755	129 (94)	1004.7 ± 56.3	0.190
IIb, III, IV	6 (4)	724.2 ± 149.0		5 (4)	783.0 ± 160.6		8 (6)	622.8 ± 117.8	
**JPS**									
1	126 (92)	1005.2 ± 56.9	0.353	123 (90)	1007.3 ± 58.2	0.811	124 (91)	1016.6 ± 57.3	0.102
2–4	11 (8)	783.8 ± 139.4		10 (7)	723.5 ± 131.1		13 (9)	705.7 ± 124.0	
**CAP**									
0–2	15 (11)	853.7 ± 109.0	0.944	17 (12)	840.5 ± 101.9	0.747	16 (12)	753.8 ± 78.8	0.719
3	122 (89)	993.3 ± 58.1		116 (85)	982.0 ± 59.9		121 (88)	982.1 ± 57.7	
**ART**									
0–3	55 (40)	927.7 ± 52.1	0.012*	63 (46)	1115.0 ± 79.7	0.067	59 (43)	923.3 ± 51.2	0.009*
4	82 (60)	879.2 ± 68.3		70 (51)	694.1 ± 41.5		78 (57)	870.3 ± 69.9	
**Necrosis**									
~ 19	70 (51)	1099.57 ± 73.6	0.031*	67 (49)	1069.3 ± 77.7	0.251	68 (50)	1089.8 ± 76.4	0.076
20 ~	67 (49)	643.1 ± 36.8		66 (48)	750.1 ± 49.6		69 (50)	741.5 ± 48.9	
**T/S ratio**									
~ 39	95 (69)	829.6 ± 43.1	0.520	85 (62)	1051.4 ± 68.5	0.069	84 (61)	880.8 ± 44.9	0.020*
40 ~	42 (31)	954.4 ± 97.0		48 (35)	619.5 ± 45.1		53 (39)	851.3 ± 82.7	

*P < 0.05 by log-rank test.

There were no cancer cells in the slides of macroscopic defined largest tumor of 4 patients; thus, we did not assess tumor regression grades of them.

## Discussion

The present study revealed that the macroscopic defined largest cancer cut surface and the largest histologic cancer slide were discordant in approximately 26% of neoadjuvant-treated pancreatic cancer cases. Furthermore, viable tumor cells were not noted in the largest macroscopically defined slides in 3%. Discrepant cases were more frequent in cases that received CT than those who received CRT. This suggests that macroscopic identification of tumors often fails to detect the largest histological slides and is influenced by many factors including treatment regimens, treatment effects, and necrosis.

Pathologic complete response or minimal residual cancer specimens have been reported to be correlated with better survival in patients with pancreatic cancer who received preoperative neoadjuvant therapy^8,13^. However, the present study failed to show an association between prognosis and tumor regression grade as determined by the CAP, Evans, and JPS grading system. This could be because the present cohort showed mild tumor regression. Only 0.7% of the present cohort had pathologic complete response, while previous studies treated with gemcitabine- and fluoropyrimidine-based chemoradiotherapy showed that the percentage of pathologic complete response ranged from 1.8 to 2.3%^14,15^. In the present study, patients were treated with gemcitabine and S-1-based chemotherapy with or without radiation, which has been widely used as neoadjuvant treatment for resectable and borderline resectable pancreatic cancer in Japan^16,17^. In western countries, FOLFIRINOX (combination of 5-fluorouracil, leucovorin, irinotecan, and oxaliplatin)^18–20^ has been widely used as neoadjuvant treatment for pancreatic cancer. The variable neoadjuvant regimens among these studies could have affected the tumor regression grade. Therefore, it is important to establish a novel evaluation method of treatment effect in accordance with various situations of neoadjuvant therapies. The ART score appears to be better suited for assessing mild tumor regression than the Evans, JPS, and CAP grading systems. Large cohort studies from various regimens of neoadjuvant therapies are warranted to further evaluate the prognostic value of pathological assessment, possibly using imaging and biomarkers.

Our previous study showed that patients who received neoadjuvant therapy (gemcitabine plus nab-paclitaxel) displayed a lower T/S ratio than patients who did not receive neoadjuvant treatment, indicating that neoadjuvant therapy induces fibrosis^5^. Therefore, in the present study, we analyzed the relationship between T/S ratio and tumor regression assessment. Contrary to our expectations, there was no significance difference in the T/S ratio between the discrepant and concordant cases. However, a low T/S ratio determined by the largest histological slide was associated with a better prognosis. Fibrosis after neoadjuvant treatment might imply that neoadjuvant therapy could maintain cytotoxic effects for a long time, resulting in improved prognosis^5^. Furthermore, a high percentage of necrosis was associated with a worse prognosis in the present study. This could be because the tumor size in the high necrosis group (33.2 ± 15.9 mm) was significantly larger than that in the low necrosis group (26.9 ± 15.5 mm).

The present study has several limitations. Patients were treated with gemcitabine and S-1-based chemotherapy with or without radiation. In addition, the number of cases was quite large in only one classification and the number of cases in the two groups differed greatly, therefore, the proportion of patients in the high regression group was biased (Incidences of Evans IIb + III + IV, JPS 1, CAP 3 and ART 0–3 were 4%, 8%, 11%, and 40%, respectively). This may be attributed to mild tumor regression in the present cohort, but it also indicates the limitation of prognostic prediction based on existing pathological grading systems. Although there are such limitations, we consider that the results of the largest histological slide are close to those of all slides from whole-tissue slices, and the largest histological slide can be satisfied to obtain an accurate evaluation of tumor regression for treated pancreatic cancer with neoadjuvant therapy. However, to create the largest histological slide, it is necessary to prepare not only the slide with the largest macroscopically defined tumor but also all slides from whole-tissue slices of the pancreatic cancer lesion.

In conclusion, the present study revealed that largest macroscopically defined or histological slides were discordant in approximately 26% of neoadjuvant-treated pancreatic cancer cases. We recommend that sufficient tissue should be obtained to identify the largest histological slides that produce more robust prognostication by tumor regression grades in pancreatic cancer tissues after neoadjuvant therapy.

## Methods

### Patients

We conducted a retrospective study using pathological tissue from surgically resected pancreatic cancer after preoperative neoadjuvant therapy. Tissue samples were obtained at the Kagawa, Juntendo, Tokai, and Tokyo Medical University Hospitals during 2009–2019. The present study included 137 patients with resectable or borderline resectable tumors. Of them, 86 were treated with chemoradiotherapy (CRT, gemcitabine, and S-1-based chemotherapy and radiation) and 51 were treated with chemotherapy (CT, gemcitabine, and S-1) before pancreatectomy (Table 1). Surgery was performed approximately 1 month after the last session of neoadjuvant chemotherapy. Preoperative resectability (resectable, borderline resectable, locally advanced, and metastatic) was determined by radiologists using computed tomography or magnetic resonance imaging. The present study was approved by the ethics committee of the Faculty of Medicine, Kagawa University (permit #2019-144), Juntendo University (#19-056), Tokai University School of Medicine (#16R273), and Tokyo Medical University (#T2018-0001). All methods were performed in accordance with the relevant guidelines and regulations. Informed written consent to use the tissues was obtained from all patients.

### Pathological evaluation of tumor regression grades

**Figure 3:**
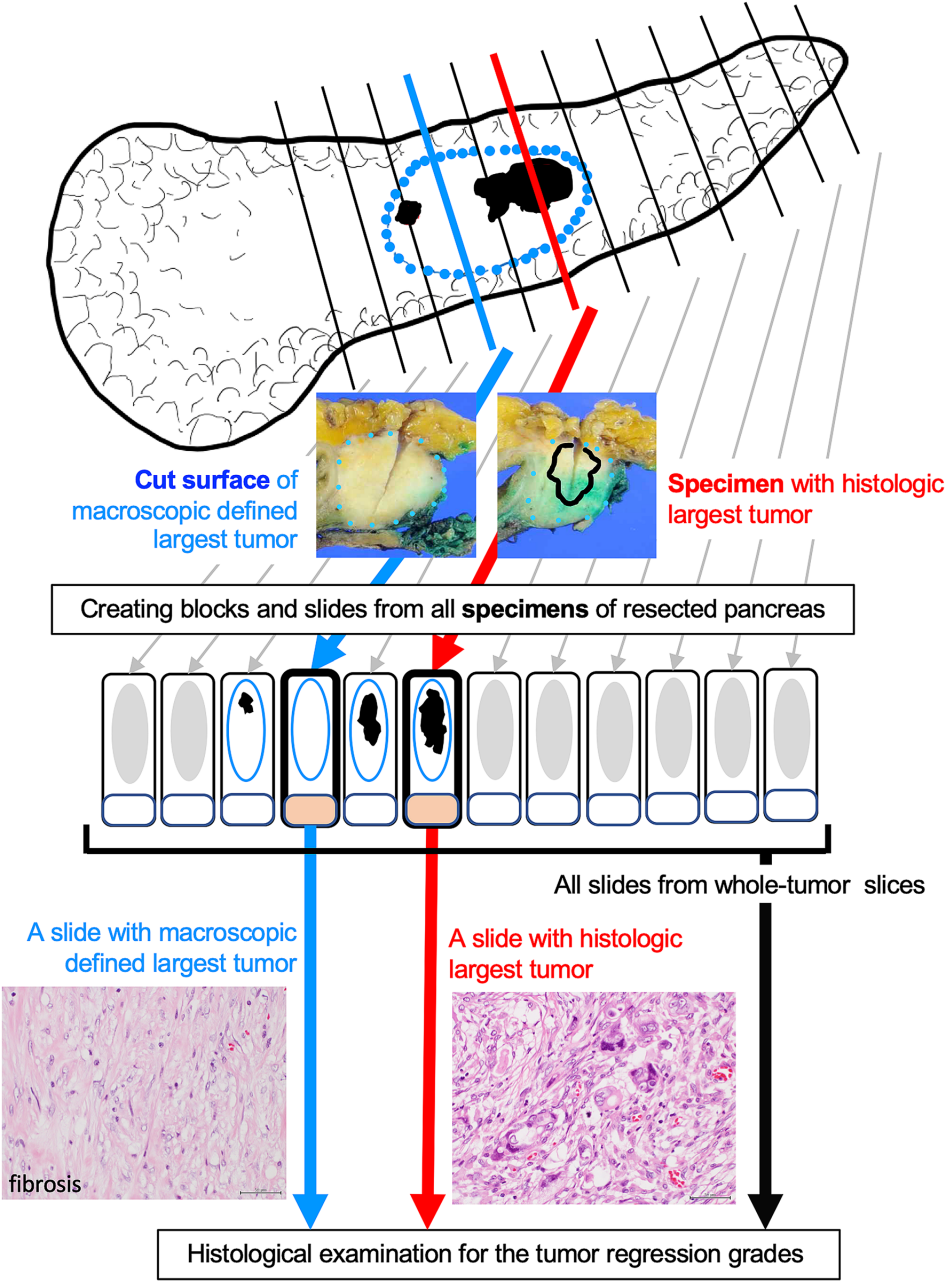
Comparison of tumor regression grades from different slides. Distal pancreatectomy sample was sliced perpendicular to the main pancreatic duct at a 5-mm interval. Tissue blocks were created from all specimens. For pathological analysis, glass slides were created from all blocks. Tumor regression grades were evaluated from a slide with the cut surface of macroscopic defined largest tumor, a slide with largest histological tumor, and all slides from whole-tumor slices. Dotted line: fibrosis after neoadjuvant therapy; black-out area or black grid-line: cancers identified histologically.

All pancreatic samples were obtained from formalin-fixed paraffin-embedded specimens. Surgically resected pancreatic tissues were fixed in 10% neutral-buffered formalin. After that, tissues were serially sliced parallel to the Kerckring fold line at approximately 5-mm intervals to both the oral- and anal-side ends in pancreatoduodenectomy cases. In distal pancreatectomy cases, tissues were serially sliced perpendicular to the long axis of the pancreas at approximately 5-mm intervals, and whole-tumor slices were sampled for histological examination (Fig. 3). Whole-tumor samples were stained with hematoxylin and eosin. Elastica van Gieson staining was performed to detect the presence of vascular invasion. More than two pathologists assessed the reporting items recommended from the 7th edition of the General Rules for the Study of Pancreatic Cancer by the JPS to establish a final assessment with consensus^9^.

Pathological evaluation was performed using all 2251 histological slides from the pancreas. Largest macroscopically defined slides were determined by the largest residual cancer area using macroscopic photographs of the cut surface of the tumor. Largest histological slides were determined by microscopy with the largest residual cancer area using microscopy. We evaluated tumor regression grades using largest macroscopically defined slides, largest histological slides, and all slides from whole-tumor slices (Fig. 3). Tumor regression grades determined by all slides were used for correct evaluation, and the prognostic utility of tumor regression grades determined by macroscopically defined or largest histological slides was compared to that of all slides. The tumor regression grades were assigned by two pathologists (M.Y. and Y.M.) according to the criteria of the JPS, Evans, CAP grading systems, and ART score.

We also determined the percentage of necrotic cancer cells and the ratio of tumor cells/stroma cells (T/S ratio) in the cancer area. Indices of the necrosis and T/S ratio percentage were determined by cut-off values of 20% and 40%, respectively, and they were divided into high and low groups. These cut-off values were determined by a ROC curve with survival or death as objective variable and the sensitivity and specificity of each values were necrosis, 55.7% and 56.6%; T/S ratio, 36.1% and 75.0%, respectively.

### Statistical analysis

Data are presented as mean ± standard deviation. Kaplan–Meier analysis was performed to analyze the relationship between overall survival and clinicopathological features. Overall survival was defined as the period from surgery to death or censor and assessed by dividing the patients into two groups for each evaluation item. Ideally, we would like to examine the correlation between each grade of Evans, JPS, CAP and ART and each item. However, the number of cases in the present study is too small to examine these correlations. Furthermore, regarding EVANs, JPS, and CAP, the number of cases was quite large in only one classification. Therefore, Evans, JPS, CAP and ART were set two groups by examination with various combinations of grades and with the most statistical differences for overall survival of patients receiving neoadjuvant therapy. As an example, in the Evans classification, combinations of I and IIa + IIb + III + IV, I + IIa and IIb + III + IV, I + IIa + IIb and III + IV, and I + IIa + IIb + III and IV were compared for overall survival, and the groups of I + IIa and IIb + III + IV with the largest difference (P value was the lowest however there was no significant inter-group difference) were set finally. The items of clinicopathological characteristics (Table 1) were also examined according to these two-group classifications. As a result, the number of cases in the two groups was set to be as close as possible. The cut-off values for necrosis and T/S ratio were determined by a ROC curve with survival or death as objective variable. Chi-square and Fisher's exact tests were used to analyze the clinicopathological features. P values were determined using the log-rank test. Multivariate correlation values between all slides and macroscopically defined or largest histological slides were determined using Pearson's product–moment correlation coefficient. Statistical analysis was performed using JMP software, version 14.3.0 (SAS Institute, Inc., NC, USA). A P value of less than 0.05 was considered statistically significant.

## Data Availability

All data generated or analysed during this study are included in this published article.

## Abbreviations

CRT: Chemotherapy and radiation before pancreatectomy
CT: Chemotherapy before pancreatectomy
JPS: The Japanese Pancreas Society grading systems
CAP: The College of American Pathologists grading systems
ART: Area of residual tumor
